# Fibronectin Extra Domain A Promotes Liver Sinusoid Repair following Hepatectomy

**DOI:** 10.1371/journal.pone.0163737

**Published:** 2016-10-14

**Authors:** Bridget Sackey-Aboagye, Abby L. Olsen, Sarmistha M. Mukherjee, Alexander Ventriglia, Yasuyuki Yokosaki, Linda E. Greenbaum, Gi Yun Lee, Hani Naga, Rebecca G. Wells

**Affiliations:** 1 Department of Medicine, Perelman School of Medicine, The University of Pennsylvania, Philadelphia, Pennsylvania, United States of America; 2 Cell and Molecular Biology Graduate Group, Perelman School of Medicine, The University of Pennsylvania, Philadelphia, Pennsylvania, United States of America; 3 Department of Physiology, Perelman School of Medicine, The University of Pennsylvania, Philadelphia, Pennsylvania, United States of America; 4 Department of Bioengineering, School of Engineering and Applied Sciences, The University of Pennsylvania, Philadelphia, Pennsylvania, United States of America; 5 Hiroshima University, Hiroshima, Japan; 6 Janssen R&D, LLC, Spring House, Pennsylvania, United States of America; 7 Department of Biology, School of Arts and Sciences, University of Pennsylvania, Philadelphia, Pennsylvania, United States of America; IDIBAPS Biomedical Research Institute, SPAIN

## Abstract

Liver sinusoidal endothelial cells (LSECs) are the main endothelial cells in the liver and are important for maintaining liver homeostasis as well as responding to injury. LSECs express cellular fibronectin containing the alternatively spliced extra domain A (EIIIA-cFN) and increase expression of this isoform after liver injury, although its function is not well understood. Here, we examined the role of EIIIA-cFN in liver regeneration following partial hepatectomy. We carried out two-thirds partial hepatectomies in mice lacking EIIIA-cFN and in their wild type littermates, studied liver endothelial cell adhesion on decellularized, EIIIA-cFN-containing matrices and investigated the role of cellular fibronectins in liver endothelial cell tubulogenesis. We found that liver weight recovery following hepatectomy was significantly delayed and that sinusoidal repair was impaired in EIIIA-cFN null mice, especially females, as was the lipid accumulation typical of the post-hepatectomy liver. *In vitro*, we found that liver endothelial cells were more adhesive to cell-deposited matrices containing the EIIIA domain and that cellular fibronectin enhanced tubulogenesis and vascular cord formation. The integrin α_9_β_1_, which specifically binds EIIIA-cFN, promoted tubulogenesis and adhesion of liver endothelial cells to EIIIA-cFN. Our findings identify a role for EIIIA-cFN in liver regeneration and tubulogenesis. We suggest that sinusoidal repair is enhanced by increased LSEC adhesion, which is mediated by EIIIA-cFN.

## Introduction

Increased secretion of extracellular matrix (ECM) proteins occurs during wound healing and is essential for tissue repair [1]. The vasculature is an important site of new ECM deposition, and adhesion of endothelial cells to this ECM promotes vascular morphogenesis [2] as well as cytokine expression and activation [3], both essential for repair. Delineating the mechanisms by which increases in matrix proteins modulate the repair and morphogenesis of the vasculature has the potential to identify new therapies that promote wound healing.

Fibronectin is one of the earliest ECM proteins deposited after injury [4, 5]. It is the product of a single gene that produces two main forms, plasma fibronectin and cellular fibronectin. Plasma fibronectin is soluble and secreted by hepatocytes while cellular fibronectin is organized into insoluble fibrils and deposited by many cell types, including myofibroblasts and endothelial cells. Cellular fibronectin undergoes alternative splicing at the C-terminus and in the region of the type III repeats; it can include two alternatively spliced type III repeats, extra domain A and extra domain B [6]. Cellular fibronectin containing these domains is known as EIIIA-cFN and EIIIB-cFN in rodents and EDA-cFN and EDB-cFN in humans; for clarity, we use the rodent nomenclature throughout this work. The expression of EIIIA-cFN and EIIIB-cFN is low in healthy adult tissues but both splice variants are prominently expressed around developing blood vessels in embryonic development [7], tumorigenesis [8] and fibrosis [9].

EIIIA-cFN binds integrins α_9_β_1_ and α_4_β_1_ in addition to the standard fibronectin integrins α_5_β_1_ and α_v_β_3_, which bind all forms of fibronectin [10]. Inclusion of the EIIIA domain in fibronectin increases cell adhesion [11] and mediates conformational changes that alter fibronectin organization in the matrix [12]. EIIIA-cFN has a role in some tissues in fibrosis and repair: EIIIA-cFN null mice have abnormal cutaneous wound healing [13] and are protected against bleomycin-induced lung fibrosis [14] and cardiac allograft fibrosis [15]. In the liver, EIIIA-cFN promotes the motility of hepatic stellate cells [16], which are the major precursors of fibrogenic myofibroblasts after injury [17]. Male EIIIA-cFN null mice are protected against thioacetamide-induced fibrosis; however, and in contrast to fibrosis in other organ systems, females with liver injury and both male and female mice with bile duct ligation have equivalent degrees of fibrosis regardless of the presence of EIIIA-cFN [16].

The role of EIIIA-cFN in the development of normal and tumor vasculature has been debated [18]. It clearly plays a role in lymphatic development: EIIIA-cFN null mice have defective lymphatic valves [19] and these defects are phenocopied by *Itga* 9 null mice, which lack the alpha subunit of integrin α_9_β_1_ [19]. In the liver, the perisinusoidal space of Disse is an initial collecting point for lymph [20], such that liver sinusoidal endothelial cells (LSECs) are adjacent to an interstitial space. Following liver injury, LSECs increase their expression of EIIIA-cFN within 12–24 hours [9].

LSECs play key roles in the sinusoidal repair process following injury [21–23]. Following partial hepatectomy, LSECs and their progenitors recruited from the bone marrow secrete soluble factors such as hepatocyte growth factor (HGF) that promote liver regeneration [22, 23]. Additionally, LSECs proliferate to increase vascularization of the regenerated liver mass during regeneration [22, 23]. The role of EIIIA-cFN in LSEC function and in the sinusoids has not been determined.

We hypothesized that EIIIA-cFN promotes sinusoidal repair and investigated this by carrying out partial hepatectomies (PHx) in EIIIA-cFN null mice and adhesion and tubulogenesis assays *in vitro* using liver endothelial cells. We demonstrate a significant delay in liver weight recovery and sinusoidal repair after partial hepatectomy in EIIIA-cFN null mice and suggest on the basis of *in vitro* studies that this is secondary to decreased LSEC adhesion.

## Materials and Methods

### Antibodies

The following antibodies were used for adhesion blockade: for integrin α_5_ subunit (clone HMα5–1, Santa Cruz Biotechnology, Santa Cruz, CA), for integrin α_v_ subunit (clone H9.2B8, Santa Cruz Biotechnology), and for integrin α_9_ subunit (antibody was supplied by Dr. Yasuyuki Yokosaki and generated in a similar manner as previously described) [24]. For immunostaining, antibodies used were against extra domain A (EIIIA) (clone IST9; Santa Cruz Biotechnology) (1:200), total fibronectin (Santa Cruz Biotechnology) (1:200), Ki-67 (Abcam) (1:200) and VE cadherin (R & D Systems, Minneapolis, MN) (1:200). For immunoblotting, antibodies used were against GAPDH (Abcam, Cambridge, MA) (1:10,000), total fibronectin (Santa Cruz Biotechnology) (1:1000), LYVE1 (Abcam)(1:100) and VEGFR3 (Abcam)(1:100).

### Mouse experiments

EIIIA-cFN^−/−^ mice, on a pure C57Bl/6 background, were obtained from a litter recovered from cryopreserved embryos from the Mutant Mouse Regional Resource Center (B6.129S4-*Fn1*^*tm1Bwg*^/Mmnc, deposited by Dr. Elizabeth George) [25]. EIIIA-cFN heterozygotes were bred to either heterozygote or null animals to generate wild type, heterozygous and null littermates. Males were used at 10–14 weeks at an average weight of 25 g while female mice were used at 14–16 weeks at an average weight of 20–23 g. There was no weight difference between wild type and EIIIA-cFN null animals. Mice were fed standard chow and were on a 12 h light/dark cycle. All animal work was performed in accordance with the U.S. Department of Health and Human Services Guide for the Care and Use of Laboratory Animals and with the approval of the University of Pennsylvania Institutional Animal Care and Use Committee; every effort was made to minimize suffering.

### Partial hepatectomy (PHx)

A two-thirds PHx was performed in mice as described previously [26]. Briefly, mice were anesthetized with isoflurane. The median and lateral lobes, comprising approximately 70% of the liver, were resected. For sham operations, the abdominal cavity was opened and the liver gently massaged without resection. At various time points after surgery, mice were euthanized and the livers removed and either fixed in 10% phosphate buffered formalin (Fisher Scientific, Pittsburgh, PA) overnight, frozen in OCT (Scigen Scientific, Gardena, CA), or snap frozen in liquid nitrogen.

### Quantitative reverse-transcription polymerase chain reaction (qRT-PCR)

Snap-frozen livers were homogenized in Trizol (Invitrogen, Carlsbad, CA) using a Bullet Blender (Next Advance, Averill Park, NY). Equal amounts of RNA from each sample were used to synthesize cDNA using Super Scriptase III (Invitrogen). Real time PCR was performed on an ABI StepOne Plus Real-Time PCR System with Fast SYBR green (Applied Biosystems, Foster City, CA), with three technical replicates obtained for each sample and a relative standard curve method used to quantify the results obtained. TATA box binding protein gene (*tbp*) was used as a housekeeping gene. Primer sequences are provided in S1 Table.

### Immunostaining

For immunofluorescence, samples frozen in OCT were cut, fixed with cold acetone (Fisher Scientific) or 10% Neutral Buffered Formalin (Fisher Scientific), and incubated with primary antibodies as above overnight at 4°C. Subsequently they were incubated with Cy5-conjugated secondary antibodies (Jackson Immunoresearch, West Grove, PA) and counterstained with the nuclear stain 4',6-diamidino-2-phenylindole (DAPI; Invitrogen). Slides were imaged with a Zeiss wide field microscope, with 4–5 representative images taken per sample. For blood vessel staining, the percentage VE-cadherin positive area per image was calculated using Image J (NIH). When photos of stained tissue are shown in figures, they are representative images, with quantification values in the middle of the range.

### Oil Red O staining

Livers frozen in OCT were cut and fixed in 10% Neutral Buffered Formalin for 4 min. Slides were placed in propylene glycol (Poly Scientific, Bay Shore, NY) for 2 min and then incubated in Oil Red O (Poly Scientific) overnight at 4°C. The slides were transferred to 85% propylene glycol solution for 1 min and counterstained with hematoxylin (Fisher Scientific). Imaging was carried out using a Nikon E600 microscope. 3–4 images were taken per section and quantification of Oil Red O positive area was done using Image J. Representative images with quantification values in the middle of the range are shown in the figures.

### Liver endothelial cell isolation and culture

Liver endothelial cells were obtained from 3–4 week old C57BL6 mice (Charles River Laboratories, Newtown, MA). Livers were harvested, diced, digested in 10 mg/ml collagenase (Worthington, Lakewood NJ) supplemented with 20 μg/ml DNase (Worthington) for 40 min at 37°C, then strained through 100 and 70 μm nylon filters. This was followed by Histodenz (Sigma, St. Louis, MO) gradient centrifugation (11%-17%) [27]. The endothelial cell/Kupffer cell fraction was plated on tissue culture plastic for 1 h to allow Kupffer cells (which readily attach to non-coated dishes) to separate from endothelial cells. The non-adherent cells were then cultured on collagen-coated plates for 3 d, and dissociated and sorted by labeling first with biotinylated anti-CD31 (1:200) (Ebioscience, San Diego, CA) and then with magnetic anti-biotin microbeads (Miltenyi Biotech, Cambridge MA). Labeled cells were separated by immunomagnetic separation and cultured on collagen-coated dishes. Cells were cultured in endothelial cell medium: Advance DMEM (Invitrogen) with 10% FBS, antibiotics, and Endothelial Cell growth supplement (Alfa Aesar, Ward Hill, MA). Cells at passages 2–4 were used for experiments and had purity > 95% as assessed by staining for von Willibrand factor. For primary integrin profiling, cells were lysed in Buffer RLT (Qiagen, Valencia, CA) and total RNA was harvested using the Qneasy Qiagen kit. qRT-PCR was performed as previously described.

### Immunofluorescence of liver endothelial cells

Liver endothelial cells at passage 3 were fixed with 4% paraformaldehyde and assessed by staining for LYVE1 and VEGFR3. Images were taken using a Zeiss wide field microscope. Immunostaining showed that these cells express LYVE 1 and VEGFR3 (Panels A-C in S1 Fig), both of which are expressed by liver endothelial cells but are absent from endothelial cells from larger vessels.

### Cloning of cellular fibronectin isoforms and generation of stable cell lines

Full-length rat fibronectin cDNA containing EIIIA, EIIIB and IIICS domains in the pLEN plasmid was provided by Dr. Pam Norton (Drexel University). To generate individual fibronectin isoforms, small portions of fibronectin bordering the EIIIA and EIIIB domains but lacking these domains were cloned into a donor plasmid by Genescript (Piscataway Township, NJ). Subsequently, the small portions were cloned into the full-length fibronectin using a restriction digestion and ligation strategy to remove the EIIIA and EIIIB domains. The fibronectin isoforms were then cloned into a pcDNA3.1 plasmid using TA cloning technology (Invitrogen). This plasmid has a V5 epitope tag and a 6XHis tag to aid in detection. The sequence of each full-length isoform was verified by double-stranded sequencing. CHO-K1 cells were transfected with each isoform and stable clones were selected using 400 μg/ml G418; these were shown to express the appropriate isoform by qRT-PCR and western blotting.

### Cell-derived matrix production

Cell-derived matrices were generated as previously described [28]. Highly-expressing stable CHO cell clones overexpressing FNs were selected and cultured on collagen coated plates or coverslips at a density of 5x10^4^ cells/cm^2^. Cultures were treated with CHO cell media supplemented with 50 μg/ml ascorbic acid (Sigma) for eight days. Matrices were decellularized on day 9 using an extraction buffer of 0.5% Triton X-100 and 20 nM NH_4_OH in PBS. Immunoblotting and immunostaining of matrices was carried out using previously established methods [28, 29].

### Adhesion assays

Adhesion assays on cell-derived matrices were performed as previously described [28]. Liver endothelial cells were first labeled with Hoechst 33342 (Invitrogen) for 15 min at 37°C. Cells were rinsed with PBS, trypsinized, counted and resuspended in endothelial cell medium. Cells were allowed to recover from trypsinization by rotating for 20 min at 37°C. For some experiments, IgG or Itga 9 blocking antibodies were added to the cell suspension at a concentration of 0.02 mg/ml. Matrices were generated as described from CHO cells overexpressing fibronectins in glass-bottom no. 1.5 dishes (MatTek Corporation, Ashland, MA). Cells were plated on matrices and incubated for 10 min at 37°C. Matrices were then rinsed with PBS and fixed with 4% paraformaldehyde (Affymetrix, Santa Clara, CA) supplemented with 0.5% sucrose for 20 min. Matrices were scanned using an EVOS Auto Cell Imaging System (Life Technologies, Carlsbad, CA). Image J was used to count the Hoescht-labeled cell nuclei.

### Transwell chemotaxis assays

Liver endothelial cells were starved overnight in 0.5% FBS/DMEM F12 (Invitrogen). Fluroblock transwell inserts (BD Biosciences, Franklin Lakes, NJ) were coated with either 0.02 mg/ml plasma fibronectin or cellular fibronectin (Sigma) on both sides for 30 min at room temperature. Cells were plated in the top chamber of transwell filters in media with 0.5% FBS and the filters were placed atop a 24-well plate with media containing 10% FBS. Inserts were incubated at 37°C for 18 h and then stained for 1 h at 37°C with calcein AM (BD Biosciences). Fluorescence intensity was measured from the bottom with a BioTek Synergy 2 microplate reader (Winooski, VT). There were three replicates for each condition.

### Cell line

TSECs, a mouse LSEC cell line, were obtained from Drs. Vijay Shah and Robert Huebert [30]. Cells were cultured in DMEM with 5% FBS, and 1% Pen/Strep with ECGS supplement (Sciencell, Carlsbad, CA).

### Tube formation assays

Twenty-four well plates were coated with Geltrex (Life Technologies) and incubated at 37°C for 30 min. 4 x 10^4^ TSECs were resuspended in DMEM (Invitrogen) that had been premixed with 0.04 mg/ml of either plasma or cellular fibronectin (Sigma) and were seeded onto each Geltrex coated well. For some experiments cells were incubated with blocking antibodies to α_5_ and α_9_ on ice for an hour before seeding onto the Geltrex coated dishes. Imaging was done using an Inverted Leica DM IRB microscope.

### Statistical Analysis

Student T test was used to determine statistical significance when two groups were compared. For more than two groups, One-Way Anova was used. Graphs were generated and statistical significance calculations were done using GraphPad Prism version 4.0 (GraphPad Software, San Diego, CA). P values were considered significant when less than or equal to 0.05.

## Results

### EIIIA-cFN is upregulated early after PHx

**Fig 1:**
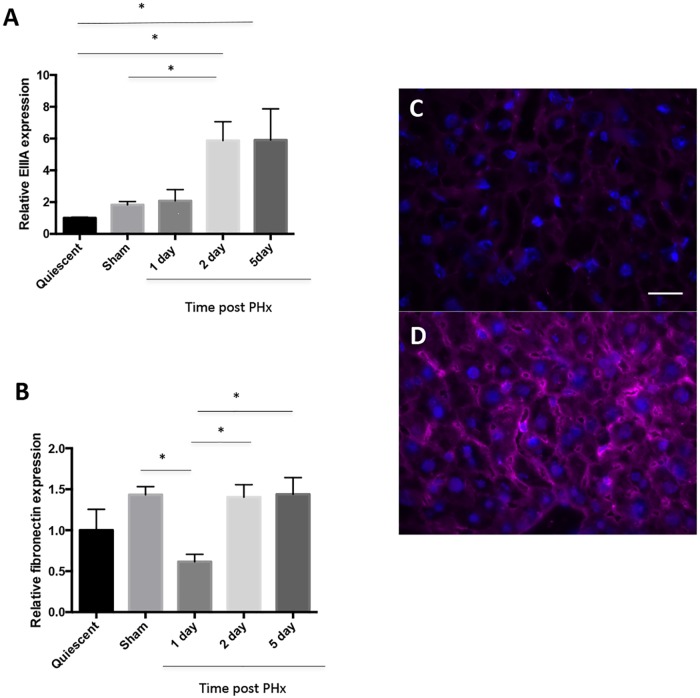
Expression of EIIIA-cFN is upregulated early after PHx. **(A, B)** Wild type mice were euthanized at days 1, 2 and 5 following PHx. mRNA transcript levels for **(A)** EIIIA-cFN and **(B)** total fibronectin were measured by qRT-PCR normalized to the expression of *tbp*. n = 3–4 mice per time point, error bars are mean +/- SEM, *p < 0.05. **(C, D)** Liver tissue from wild type **(C)** control mice or **(D)** mice at day 2 after PHx was stained for EIIIA-cFN (magenta) and with DAPI (blue). Scale bar, 20 μm.

In order to study the role of EIIIA-cFN in the sinusoidal response to injury, we first characterized the dynamics of its expression in wild type mice after 70% PHx. In agreement with the findings of Caputi et al. [31], qRT-PCR of whole liver lysates from wild type mice showed that mRNA expression of EIIIA-cFN was markedly upregulated following PHx, with a five-fold increase in comparison to quiescent livers at 48 h following PHx (Fig 1A). Interestingly, total fibronectin mRNA expression was transiently decreased at day 1 following PHx but reached pre-PHx expression by day 2 following PHx (Fig 1B); there was thus a disproportionate increase in EIIIA-cFN. Immunostaining of liver sections at day 2 after PHx showed an increase in deposition of EIIIA-cFN in a sinusoidal pattern (Fig 1C and 1D and S2A and S2B Fig).

### Delayed recovery from PHx in EIIIA-cFN null mice

**Fig 2:**
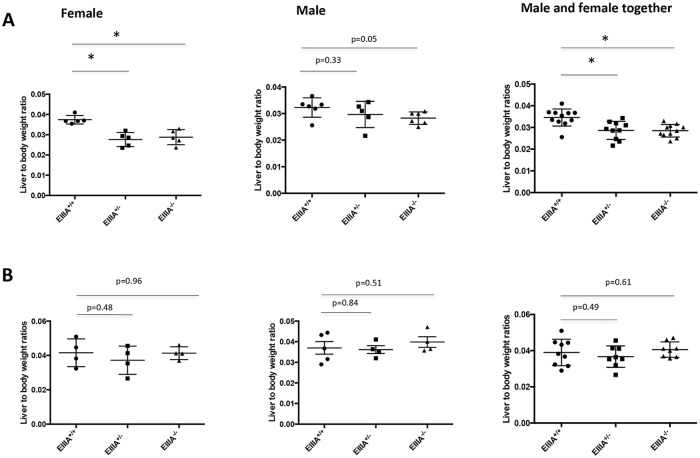
EIIIA-cFN null mice have delayed recovery of liver-to-body weight ratios following PHx. Mice were euthanized at days 2 and 5 following PHx. Liver-to-body weight ratios were measured for EIIIA-cFN null, heterozygous and wild type littermates. **(A)** Day 2 time point: EIIIA^+/+^ n = 11 (6 males, 5 females), EIIIA^+/-^ n = 10 (5 males, 5 females), EIIIA^-/-^ n = 11 (6 males, 5 females). **(B)** Day 5 time point: EIIIA^+/+^ n = 8 (4 males, 4 females), EIIIA^+/-^ n = 8 (4 males, 4 females), EIIIA^-/-^ n = 8 (4 males, 4 females). Quantification = mean +/- SD, * p<0.05.

We investigated the importance of EIIIA-cFN expression in liver regeneration by carrying out PHx in wild type, heterozygote and EIIIA-cFN null animals of both sexes. We found that mice deficient in EIIIA-cFN, especially female null and heterozygote animals, had delayed recovery of liver-to-body weight ratios following PHx (Fig 2). At day 2 following PHx, we found that EIIIA-cFN null and heterozygote mice had a mean 17% decrease in liver-to-body weight ratios in comparison to wild type littermates (Fig 2A). Female heterozygous and EIIIA-cFN null mice had a mean 27% decrease in liver-to-body weight ratio in comparison to wild type controls while male mice had a 12% decrease in liver to body weight ratios (Fig 2A). The lower liver-to-body weight ratios seen in female mice were due to decreased liver weights, not to changes in body weight, as total body weights were similar between null and wild type littermates (S3A and S3B Fig).

Analysis of livers for the expression of proliferation marker Ki-67 demonstrated a modest decrease in staining in female EIIIA-cFN null mice in comparison to wild type littermates while results were similar between male wild type and EIIIA-cFN null mice (S4A and S4B Fig). At day 5 following PHx, there was no significant difference in the liver-to-body weight ratios between EIIIA-cFN null and wild type controls for either sex (Fig 2B); liver weights and body weights were also similar for EIIIA-cFN null mice and wild type littermates of both sexes (S5 Fig) as was Ki-67 staining of tissue sections (S4C and S4D Fig). Collectively, these data demonstrate that EIIIA-cFN expression promotes recovery of liver weight following PHx, especially in female mice.

Consistent with the lower liver-to-body weight ratios after PHx in female EIIIA-cFN null mice, we found that female null mice had a slight decrease in survival following PHx compared to wild type controls (S6A Fig), although this did not reach statistical significance; we were unable to identify an obvious cause. Survival following PHx in male EIIIA-cFN null mice was comparable to wild type littermates. The expression of hepatocyte growth factor (HGF), a mitogen that promotes regeneration [22, 23] was similar in EIIIA-cFN null and wild type mice at day 2 following PHx (S6B Fig). Likewise, the expression of Angiopoietin 2, a growth factor involved in the regulation of liver regeneration, was similar between EIIIA null and wild type mice (S6C Fig).

**Fig 3:**
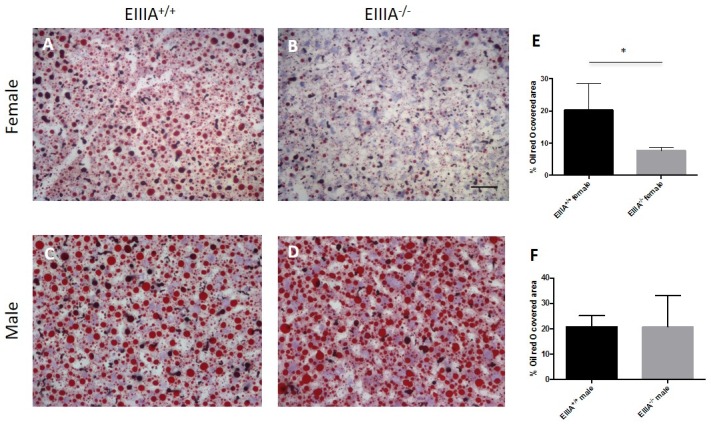
Female EIIIA-cFN null mice have decreased accumulation of intracellular lipids at day 2 after PHx. Frozen liver sections from animals at day 2 following PHx were stained with Oil Red O; lipid droplets (red), hematoxylin (blue). Oil Red O staining was decreased in female EIIIA-cFN null mice **(B)** in comparison to wild type littermates **(A)**. Staining for males was comparable between EIIIA-cFN null **(D)** and wild type littermates **(C). (E, F)** Quantification of percent Oil Red O covered area, mean +/- SD, * p<0.05. Scale bar, 50 μm. (EIIIA^+/+^ = 10; 5 female, 5 male; EIIIA^-/-^ = 10; 4 female, 6 male).

PHx results in production of cytokines that promote release of fatty acids by peripheral adipose tissue; these fatty acids circulate, are taken up by hepatocytes, and are stored as triglycerides in lipid droplets, providing energy for liver regeneration [32, 33]. Given that EIIIA-cFN null mice had delayed recovery of liver-to-body weight ratios following PHx, we investigated liver lipid accumulation after PHx by Oil Red O staining. Both wild type and EIIIA-cFN male null mice had comparable staining with Oil Red O, but female nulls had about a three-fold decrease in mean Oil Red O staining in comparison to wild type controls (Fig 3A–3F). This suggests that lipid uptake in the female nulls was inadequate for normal levels of liver regeneration. Analysis of tissue sections at day 5 following PHx demonstrated decreased accumulation of Oil Red O in comparison to day 2 samples but was comparable between EIIIA-cFN null mice and wild type mice of both sexes (S7 Fig).

### Sinusoidal abnormalities after PHx in EIIIA-cFN null mice

**Fig 4:**
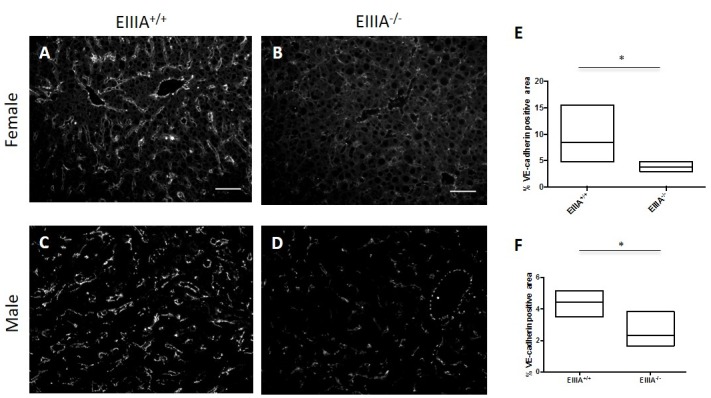
EIIIA promotes expression of the vasculature marker VE-cadherin following PHx. Frozen liver sections taken at day 2 after PHx were stained for VE-cadherin (white). Livers from female and male wild type mice showed increased staining for VE-cadherin **(A, C)** compared to livers from EIIIA-cFN null mice **(B, D)**. Scale bar, 50 μm. **(E, F)** Quantification = minimum to maximum % VE cadherin positive area measurements with line at mean, EIIIA^+/+^ (n = 10; 5 female, 5 male); EIIIA^-/-^ (n = 10; 5 female, 5 male). * p<0.05.

LSECs and their progenitors play a key role in early liver regeneration [22, 23]. Given the differences in liver-to-body weight ratios in EIIIA-cFN null mice vs. their wild type littermates at day 2 following PHx, we sought to determine whether there were differences in the sinusoids of these mice. We stained male and female liver tissue sections for VE-cadherin, a major endothelial junction protein required for junction integrity [34] and found that the percent VE-cadherin-positive area was decreased significantly in EIIIA-cFN null mice of both sexes compared to wild type littermate controls (Fig 4). These data strongly suggest that there are defects in the integrity of the sinusoids in EIIIA-cFN null mice at day 2 following PHx. Analysis of sinusoids at day 5 following PHx demonstrated comparable percent VE-cadherin-positive area between EIIIA-cFN null mice and wild type mice of both sexes (S8 Fig). Our results therefore suggest that there is a delay in the recovery of the vascular network following PHx in EIIIA-cFN null mice, although they eventually catch up to the wild type mice. This could represent either a delay in expression of key LSEC markers, or (more likely) a decrease in LSEC adhesion. Expression of vascular endothelial growth factor A (VEGFA), a major pro-angiogenic growth factor in liver regeneration at day 2 following PHx, was comparable between EIIIA-cFN null and wild type littermates (S9A, S9B and S9E Fig) as was the expression of its receptor VEGFR2 (S9C, S9D and S9F Fig).

### EIIIA-cFN, via integrin α_9_β_1_, enhances liver endothelial cell adhesion and tubulogenesis in vitro

**Fig 5:**
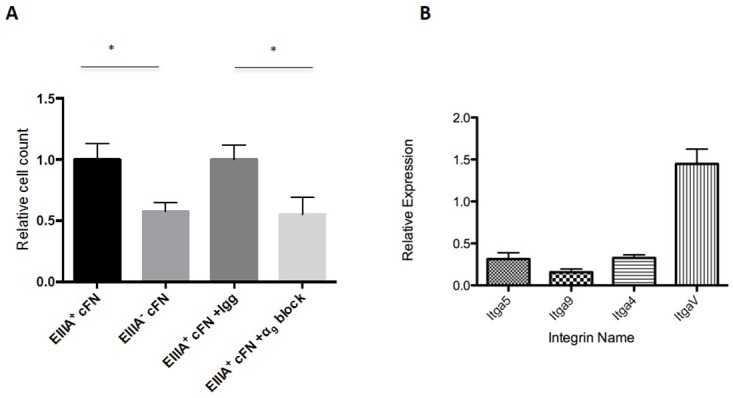
Liver endothelial cells are more adhesive to cell-deposited matrices containing EIIIA. **(A)** Mouse liver endothelial cells were allowed to bind to cell-deposited matrices with or without EIIIA-cFN, and with or without blocking antibodies to the integrin α_9_ subunit. Graph shows number of cells adhering to either EIIIA^+^ or EIIIA^-^ matrix, normalized to cells adhering to EIIIA^+^ matrix. Images shown represent 3 independent experiments, mean +/- SEM. **(B)** Integrin profiling of primary liver endothelial cells. RNA was collected from primary liver endothelial cells at day 8 after isolation. qRT-PCR shows expression of EIIIA-cFN and total FN binding integrins α_4_, α_5_, α_9_, and α_V_ relative to the housekeeping gene ribosomal protein S12.

Vascular morphogenesis requires endothelial cell adhesion to the ECM [35]. Liver regeneration also requires mobilization of LSECs from the bone marrow and their adhesion to the sinusoids, thus a potential mechanism for discontinuous sinusoids after PHx in EIIIA-cFN null mice is decreased adhesion of LSECs to matrix lacking EIIIA-cFN [23]. We tested this in vitro by generating complex cell-deposited matrices from CHO cells overexpressing EIIIA-cFN (S10A Fig). We confirmed similar deposition of total fibronectin on the matrices with or without EIIIA (S10B and S10C Fig) and performed adhesion assays on these matrices using mouse liver endothelial cells at passage 3 after isolation. Adhesion assays showed that 50% more cells adhered to EIIIA-cFN plus matrices than EIIIA-cFN minus matrices (Fig 5A). In order to determine the mechanism by which EIIIA-cFN enhances adhesion, we analyzed the integrin expression profile of these cells using qRT-PCR. We found that they express the α_5_ and α_V_ integrin subunits (subunits of the classical fibronectin binding integrins α_5_β_1_ and α_V_β_1_) as well as the α_4_ and α_9_, subunits of the EIIIA domain-binding integrins α_4_β_1_ and α_9_β_1_ (Fig 5B) [10]. When we blocked the activity of α_9_β_1_, an integrin that is particularly important for endothelial cell adhesion and lymphatic organization [36], we found that adhesion to EIIIA-cFN was significantly reduced (Fig 5A). Collectively, these data demonstrate that integrin α_9_β_1_ plays a major role in liver endothelial cell adhesion to EIIIA-cFN.

**Fig 6:**
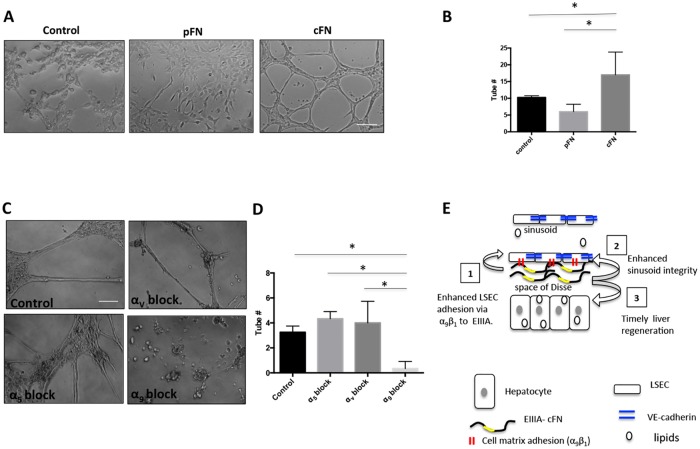
Cellular fibronectin enhances tubulogenesis and vascular cord formation in vitro. **(A)** In a tubulogenesis assay, TSECs were premixed with cellular fibronectin (cFN), plasma fibronectin (pFN), or no fibronectin (control) and cultured on Geltrex for 18 h. TSECs incubated with cellular fibronectin had enhanced tubulogenesis and aligned to form cords that were absent in the plasma fibronectin and control conditions. **(B)** Quantification shows mean tube number +/- SD, *p<0.05. Data from three independent experiments, each with three technical replicates. **(C)** TSECs were incubated with blocking antibodies to α_5_, α_9_, or α_V_ or with control IgG for 1 h, premixed with cellular fibronectin and cultured for 18 h on Geltrex. **(D)** Quantification shows mean tube number +/- SD, *p<0.05. Data from three independent experiments, each with three technical replicates. **(E)** A model for the role of EIIIA-cFN in sinusoidal repair and liver regeneration. We propose that 1) the presence of EIIIA-cFN promotes LSEC adhesion, which leads to 2) enhanced sinusoid integrity and hepatocyte lipid accumulation, which in turn results in 3) timely regeneration following PHx.

To model defects in the sinusoids that arise from the absence of EIIIA-cFN-mediated repair, we performed tubulogenesis assays using the TSEC line of mouse LSECs. We found that mixing TSECs with cellular fibronectin, which contains EIIIA-cFN, promoted tubulogenesis (Fig 6A and 6B). More importantly, unlike cells mixed with plasma fibronectin, which lacks the EIIIA and EIIIB domains, cells mixed with cellular fibronectin aligned into solid, cord-like, polygonal structures, suggesting an important role for cellular fibronectin in vascular morphogenesis. Chemotaxis of liver endothelial cells measured using transwell chemotaxis assays, however, was comparable on cellular fibronectin and plasma fibronectin (S11 Fig). We next inhibited the function of fibronectin- and EIIIA-domain-binding integrins using blocking antibodies and performed tubulogenesis assays. We found that inhibition of the α_9_ subunit specifically abrogated tubulogenesis; blocking α_5_ and α_v_ had no significant effect (Fig 6C and 6D). Overall, these data suggest that cellular fibronectins enhance tubulogenesis and that the integrin α_9_ subunit is required for this process.

## Discussion

In this study, we examined the role of EIIIA-cFN in remodeling of the liver sinusoidal vasculature following hepatectomy. We demonstrate that EIIIA-cFN mice, particularly females, have delayed recovery of liver-to-body weight ratios, decreased accumulation of hepatocyte lipid droplets, and defects in sinusoidal repair following PHx. In vitro, liver endothelial cells were more adhesive to matrices that incorporated EIIIA-cFN, and EIIIA-containing cellular fibronectin promoted liver endothelial cell tubulogenesis; the integrin α_9_β_1_ was required for these processes. These findings demonstrate that changes in the ECM can significantly affect sinusoidal function and identify a new role for EIIIA-cFN as an important regulator of sinusoidal integrity at early time points following PHx.

The finding that EIIIA-cFN-containing matrices enhance liver endothelial cell adhesion is consistent with a previously demonstrated role for EIIIA-cFN in cell adhesion [11, 37]. Our cell-deposited matrices incorporate full length EIIIA-cFN, organized by cells, and allow us to preserve organizational and conformational characteristics of the matrix as well as relevant matrix-to-matrix interactions that may be important for downstream cell signaling [38, 39], offering significant advantages over the use of isolated EIIIA domain polypeptides for adhesion studies. The observation that α_9_β_1_ is crucial for adhesion of liver endothelial cells to EIIIA-cFN-containing matrices correlates with known roles of α_9_β_1_ in mediating cell adhesion to the EIIIA domain.

One study previously reported that EIIIA-cFN and EIIIB-cFN are not required for physiological and pathological angiogenesis (or are able to compensate for each other) [18]. However, that study focused on the neovascularization of retinas and pancreatic tumors while our analysis focused on the liver sinusoids, which are unique vessels lined by a unique endothelial cell population with both vascular and lymphatic features. LSECs differ from other endothelial cells both morphologically and functionally [40, 41], and their lymphatic features suggest a potential role for EIIIA-cFN, which plays an important role in lymphatic development. Our system differs from previously studied models in other important ways. In the PHx model of injury, EIIIA-cFN but not EIIIB-cFN is upregulated early [31], suggesting that there is no compensatory increase in EIIIB-cFN. Additionally, we observed in this study and in a previous study of liver fibrosis [16] in EIIIA-cFN null mice that there are differences between males and females, which has not been observed in other studies of EIIIA-cFN and the vasculature. Thus, there are several reasons to believe that EIIIA-cFN may have a distinct role in the liver.

The finding that female EIIIA-cFN null mice have decreased accumulation of lipids and defects in sinusoidal repair raises an important question about the relationship between the integrity of the sinusoids and the efficient uptake of fatty acids. Mobilization of peripheral lipid stores and lipid accumulation in hepatocytes play a key role in providing energy for regeneration [33, 34], but little is known about the role of the sinusoidal endothelium in transporting fatty acids to hepatocytes. In the heart, lipoprotein lipase is positioned at the luminal surface of endothelial cells and is required for delivery of lipids across the endothelium to cardiomyocytes [42]. Lipoprotein lipase is likewise on the luminal surface of the capillary endothelium [43], where it may play a similar role. We hypothesize that reduced adhesion of LSECs results in a reduction (although not complete loss) of effective lipid transport. The slight decrease in regeneration at day 2 in the setting of normal lipid stores and decreased sinusoidal integrity in males suggests that the role of EIIIA-cFN and the relationship between LSECs and lipids is multifaceted. It will be important in the future to explore the role of LSECs and their lipoprotein lipases in lipid uptake and delivery to hepatocytes.

Our work is consistent with other studies showing that rapid reorganization of the ECM after PHx is important for liver regeneration [44]. Scaffolds of ECM and growth factor placed in regenerating rat livers enhance hepatocyte proliferation [45]. Ablation of Integrin-linked Kinase (involved in transmission of signals from the ECM) in hepatocytes impairs termination of regeneration and results in increased liver mass [46], with delayed increases in proliferation. The findings of these studies are in agreement with ours, which demonstrate that ECM changes are generally important for liver regeneration.

There were striking differences in our study between female and male EIIIA-cFN null mice, with females post PHx demonstrating lower liver-to-body weight ratios, decreased lipid accumulation, and defects in the sinusoidal vasculature. A previous study demonstrated that catecholamines are involved in regulating liver regeneration; in female mice, regeneration is regulated by both α and β adrenergic pathways while the α adrenergic pathway only was shown to be involved in liver regeneration in males and ovariectomized females [47]. It has also been shown that activation of transforming growth factor-β from the latent state varies in females and males; this was potentially the cause of differences in liver fibrosis susceptibility between male and female EIIIA-cFN null mice [16]. Future experiments should examine whether these pathways are involved in the phenotype we observe in EIIIA-cFN male vs. female null mice. Regardless, our findings of sex differences in both PHx and liver fibrosis models suggest that these studies should in the future include both male and female mice.

We propose a model whereby increased expression of EIIIA-cFN following PHx promotes liver sinusoidal repair and liver weight recovery in part by enhancing adhesion of LSECs (although our data are also consistent with decreased VE-cadherin expression and function), which in turn promotes lipid accumulation (Fig 6E). This model is supported by tubulogenesis assays showing that cellular fibronectin promotes tubulogenesis.

## Supporting Information

S1 Fig Analysis of liver endothelial cells via immunostaining. At passage 3, prior to utilization of liver endothelial cells for experiments, cells were stained for VEGFR3 and LYVE1, markers present on LSECs but absent from endothelial cells from larger vessels. **(A)** No primary control, **(B)** VEGFR3 (red), **(C)** LYVE1 (red). DAPI nuclear stain (blue). Scale bar 50 μm.
(TIF)

S2 Fig Expression of EIIIA-cFN is upregulated early after PHx in wild type mice. Wild type and EIIIA-cFN null mice were euthanized at day 2 following PHx. Liver tissue sections were stained for EIIIA-cFN (magenta) and with DAPI (blue). **(A)** Wild type mice, **(B)** EIIIA null mice. Scale bar, 50 μm.
(TIF)

S3 Fig EIIIA null mice, especially females, show delayed recovery of liver weights but comparable body weights as wild type littermates. Mice were euthanized at day 2 following PHx. Liver and body weights at the day 2 time point: EIIIA^+/+^ (n = 11; 5 females, 6 males), EIIIA^+/-^ (n = 10; 5 females, 5 males), and EIIIA^+/-^ (n = 11; 5 females, 6 males) mice. Liver weights at day 2 after PHx are lower in EIIIA-cFN null mice, specifically females, while body weights are comparable.
(TIF)

S4 Fig Hepatocyte proliferation after PHx measured by Ki-67 staining. Liver sections of wild type and EIIIA null mice were stained at day 2 **(A,B)** and 5 **(C,D)** following PHx. Immunostaining shows modest decreases in Ki-67 positive nuclei in female EIIIA-cFN null mice in comparison to their wild type littermates while staining in male EIIIA-cFN null and wild type mice is comparable. **(A,B)** EIIIA^+/+^ (n = 5; females, 4 males), EIIIA^-/-^ (n = 4; females, 4 males). **(C,D)** At day 5, EIIIA-cFN null mice and wild type littermates have comparable Ki-67 staining. EIIIA^+/+^ (n = 4; females, 4 males), EIIIA^-/-^ (n = 4; females, 4 males).
(TIF)

S5 Fig Comparable liver and body weights in EIIIA null mice and wild type littermates after PHx. Mice were euthanized at day 5 following PHx. **(A)** Liver and **(B)** body weights are shown for EIIIA^+/+^ (n = 8; 4 females, 4 males), EIIIA^+/-^ (n = 8; 4 females, 4 males), and EIIIA^-/-^ mice (n = 8; 4 females, 4 males).
(TIF)

S6 Fig Female EIIIA null mice show a trend towards decreased survival following PHx. **(A)** Survival graphs for EIIIA-cFN null and wild type littermates following PHx. EIIIA^+/+^ (n = 20; 10 females, 10 males), EIIIA^-/-^ (n = 25; 13 females, 12 males), females p = 0.26, males p = 0.66. Mice were only included in the survival analysis if they had no operative complications during surgery or in the 8 h following PHx. **(B)** mRNA transcript levels for HGF and **(C)** Angiopoietin 2 (Ang 2), measured by qRT-PCR, normalized to the expression of *tbp* for livers at day 2 post PHx. Sham = 7, EIIIA^+/+^ = 11 (5 females, 6 males), EIIIA^-/-^ (n = 12; 5 females, 7 males).
(TIF)

S7 Fig Comparable Oil Red O staining between EIIIA-cFN null mice and wild type littermates at day 5 following PHx. Frozen liver sections were stained at day 5 following PHx. Lipid droplets (red), hematoxylin (blue). Oil Red O staining was comparable in EIIIA-cFN null mice of both sexes **(B, D)** in comparison to wild type littermates **(A, C)**. Scale bar, 50 μm. Quantification of percent Oil Red O covered area, mean +/- SD, for female mice **(E)** and male mice **(F)**. (EIIIA^+/+^ = 8; 4 males, 4 females; EIIIA^-/-^ = 7; 3 males, 4 females).
(TIF)

S8 Fig Comparable expression of VE-cadherin at D5 following PHx in EIIIA-cFN null mice and wild type littermates. Frozen liver sections taken at day 5 after PHx were stained for VE-cadherin (white). Wild type livers from female and male mice showed comparable staining for VE-cadherin **(A, C)** compared to livers from EIIIA-cFN null mice **(B, D)**. Scale bar, 50 μm. **(E, F)** Quantification = minimum to maximum % VE-cadherin-positive area measurements with line at mean, EIIIA^+/+^ (n = 8; 4 female, 4 male), EIIIA^-/-^ (n = 8; 4 females, 4 males).
(TIF)

S9 Fig Comparable expression of VEGFA and VEGFR2 in EIIIA-cFN null mice and wild type littermates. Total RNA was purified from liver lysates at day 2 following PHx and the expression of **(A, B)** VEGFA and **(C, D)** VEGFR2 was determined by qRT-PCR and normalized to the expression of *tbp*. Sham = 7, EIIIA^+/+^ (n = 11, 5 female, 6 male), EIIIA^-/-^ (n = 12, 5 female, 7 male).
(TIF)

S10 Fig Generation of decellularized matrices from CHO cells overexpressing cellular fibronectins. **(A)** CHO cells overexpressing EIIIA+ and EIIIA- cFNs were cultured for 8 days with ascorbic acid as shown in schema. **(B)** Immunostaining of matrices for total fibronectin. **(C)** Immunoblot of proteins from decellularized EIIIA+ and EIIIA- matrices, probed for total fibronectin. Loading control GAPDH.
(TIF)

S11 Fig TSECs have comparable chemotaxis on cellular and plasma fibronectin. TSECs were plated on cFN- or pFN-coated transwell inserts for 19 hours. Cells were then stained with calcein and mean fluorescence intensity on the underside of the filter was measured. Graph shows mean +/- SD.
(TIF)

S1 Table qRT-PCR primer sequences. Primer pairs were designed using Integrated DNA Technologies SciTools Real-Time PCR software.
(DOCX)
