# Supplementary figures and images for: Fibronectin Extra Domain A Promotes Liver Sinusoid Repair following Hepatectomy
Source: PLoS One. 2016 Oct 14;11(10):e0163737. doi: 10.1371/journal.pone.0163737 (PMC5065221; doi:10.1371/journal.pone.0163737)

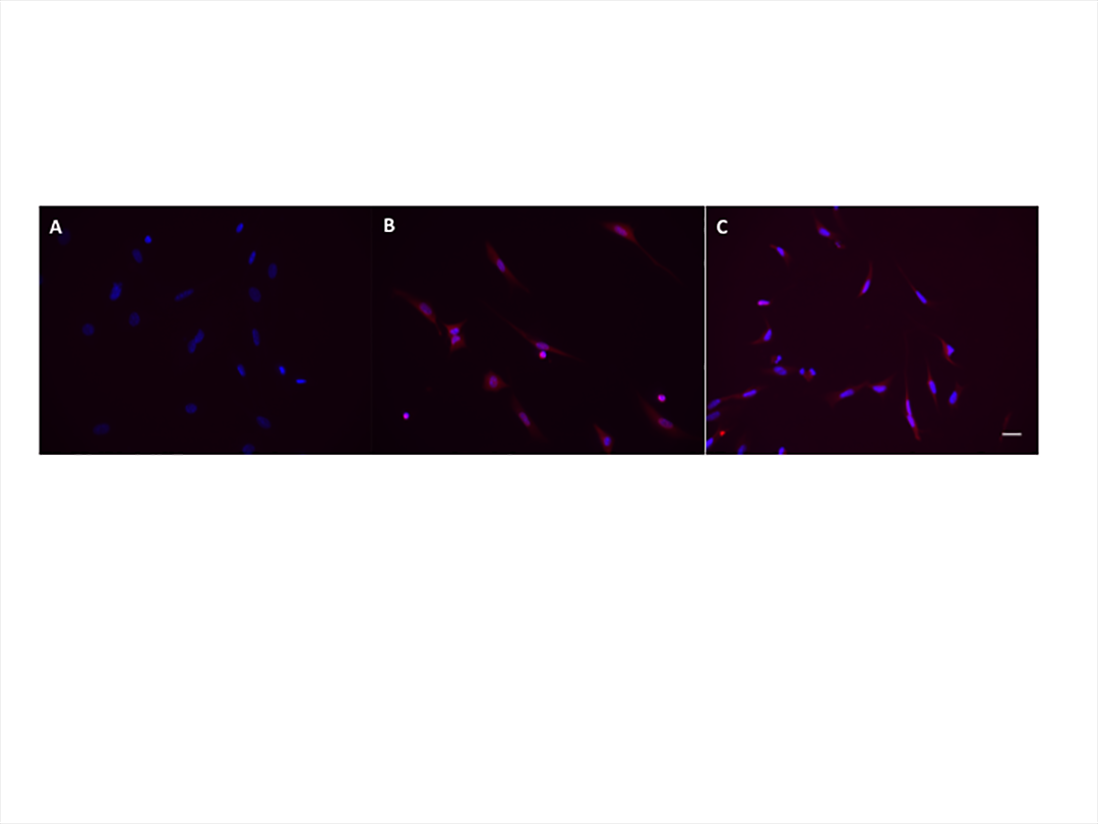

Supplement: S1 Fig — At passage 3, prior to utilization of liver endothelial cells for experiments, cells were stained for VEGFR3 and LYVE1, markers present on LSECs but absent from endothelial cells from larger vessels. (A) No primary control, (B) VEGFR3 (red), (C) LYVE1 (red). DAPI nuclear stain (blue). Scale bar 50 μm. (TIF) [file pone.0163737.s001.tif]

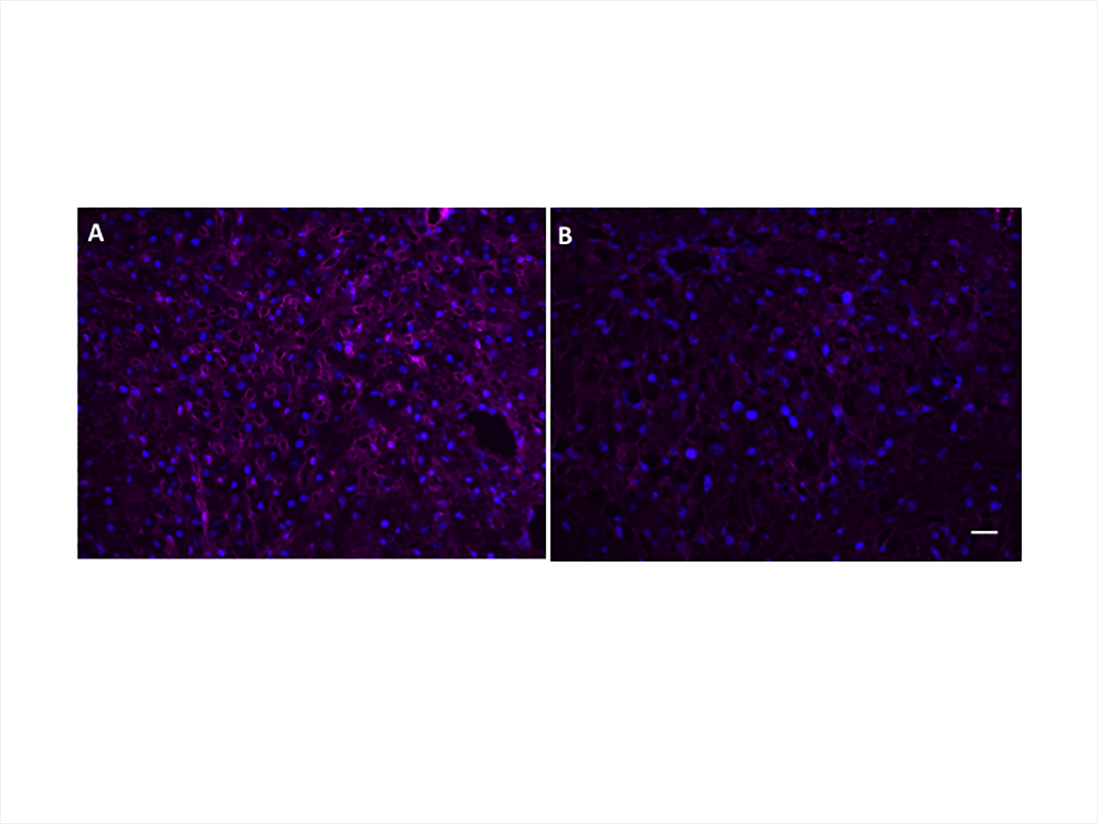

Supplement: S2 Fig — Wild type and EIIIA-cFN null mice were euthanized at day 2 following PHx. Liver tissue sections were stained for EIIIA-cFN (magenta) and with DAPI (blue). (A) Wild type mice, (B) EIIIA null mice. Scale bar, 50 μm. (TIF) [file pone.0163737.s002.tif]

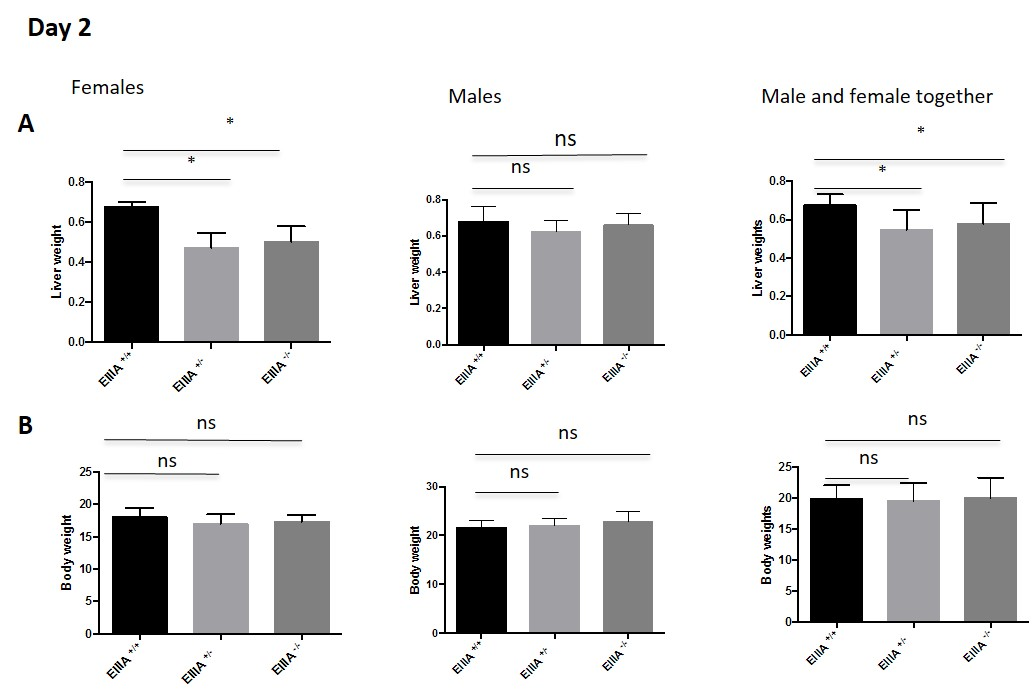

Supplement: S3 Fig — Mice were euthanized at day 2 following PHx. Liver and body weights at the day 2 time point: EIIIA+/+ (n = 11; 5 females, 6 males), EIIIA+/- (n = 10; 5 females, 5 males), and EIIIA+/- (n = 11; 5 females, 6 males) mice. Liver weights at day 2 after PHx are lower in EIIIA-cFN null mice, specifically females, while body weights are comparable. (TIF) [file pone.0163737.s003.tif]

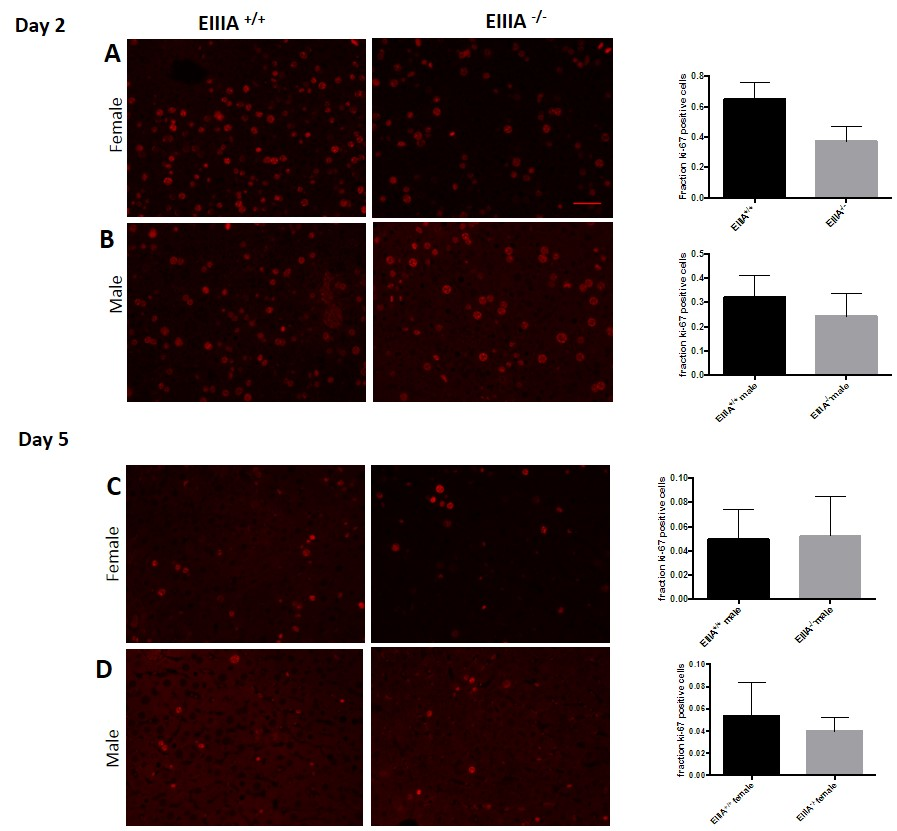

Supplement: S4 Fig — Liver sections of wild type and EIIIA null mice were stained at day 2 (A,B) and 5 (C,D) following PHx. Immunostaining shows modest decreases in Ki-67 positive nuclei in female EIIIA-cFN null mice in comparison to their wild type littermates while staining in male EIIIA-cFN null and wild type mice is comparable. (A,B) EIIIA+/+ (n = 5; females, 4 males), EIIIA-/- (n = 4; females, 4 males). (C,D) At day 5, EIIIA-cFN null mice and wild type littermates have comparable Ki-67 staining. EIIIA+/+ (n = 4; females, 4 males), EIIIA-/- (n = 4; females, 4 males). (TIF) [file pone.0163737.s004.tif]

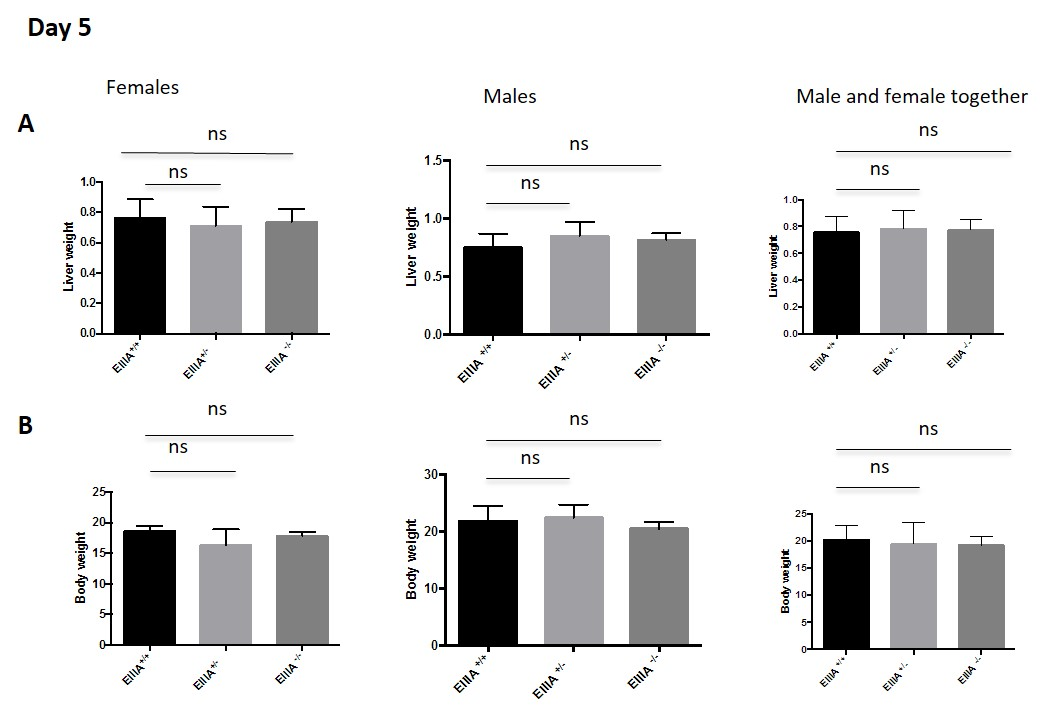

Supplement: S5 Fig — Mice were euthanized at day 5 following PHx. (A) Liver and (B) body weights are shown for EIIIA+/+ (n = 8; 4 females, 4 males), EIIIA+/- (n = 8; 4 females, 4 males), and EIIIA-/- mice (n = 8; 4 females, 4 males). (TIF) [file pone.0163737.s005.tif]

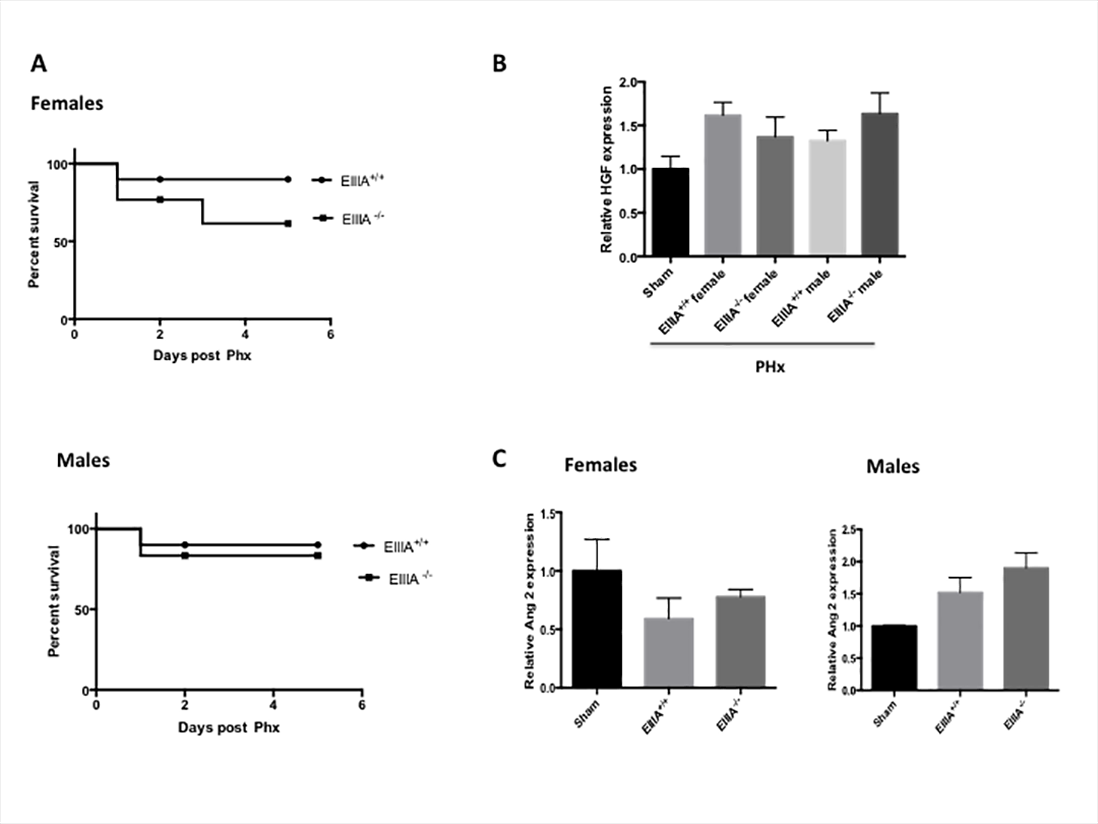

Supplement: S6 Fig — (A) Survival graphs for EIIIA-cFN null and wild type littermates following PHx. EIIIA+/+ (n = 20; 10 females, 10 males), EIIIA-/- (n = 25; 13 females, 12 males), females p = 0.26, males p = 0.66. Mice were only included in the survival analysis if they had no operative complications during surgery or in the 8 h following PHx. (B) mRNA transcript levels for HGF and (C) Angiopoietin 2 (Ang 2), measured by qRT-PCR, normalized to the expression of tbp for livers at day 2 post PHx. Sham = 7, EIIIA+/+ = 11 (5 females, 6 males), EIIIA-/- (n = 12; 5 females, 7 males). (TIF) [file pone.0163737.s006.tif]

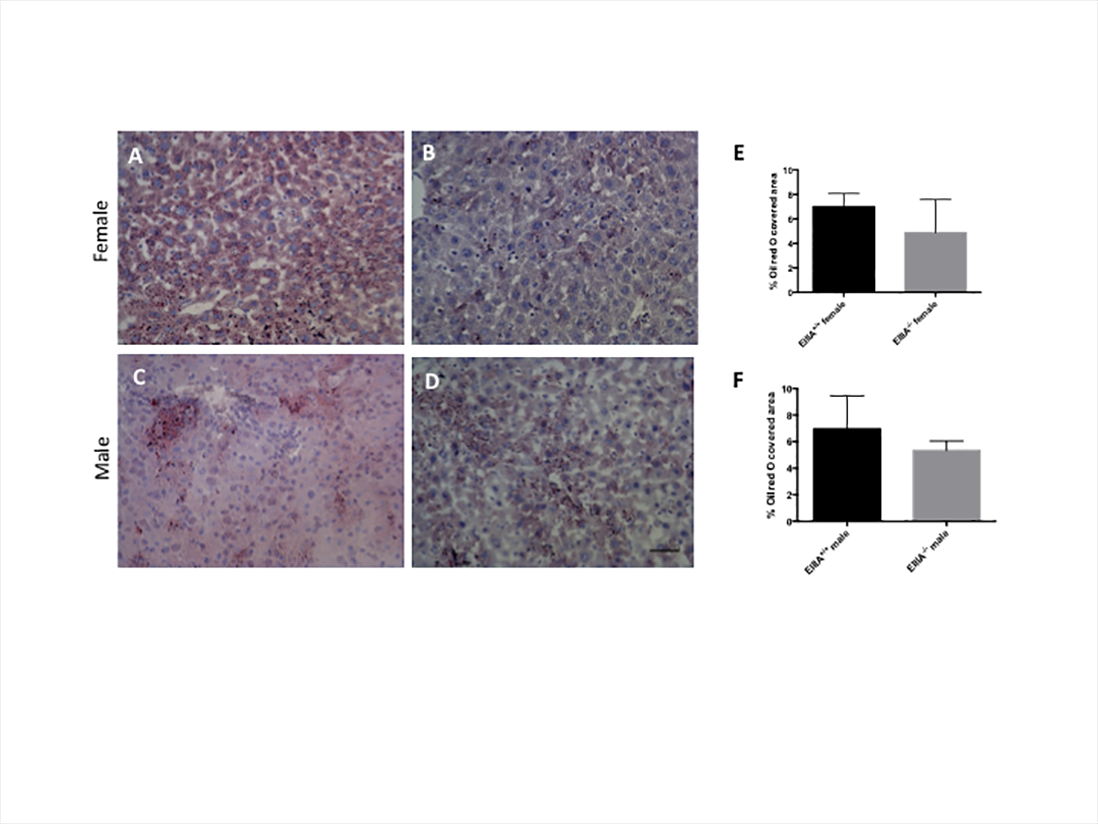

Supplement: S7 Fig — Frozen liver sections were stained at day 5 following PHx. Lipid droplets (red), hematoxylin (blue). Oil Red O staining was comparable in EIIIA-cFN null mice of both sexes (B, D) in comparison to wild type littermates (A, C). Scale bar, 50 μm. Quantification of percent Oil Red O covered area, mean +/- SD, for female mice (E) and male mice (F). (EIIIA+/+ = 8; 4 males, 4 females; EIIIA-/- = 7; 3 males, 4 females). (TIF) [file pone.0163737.s007.tif]

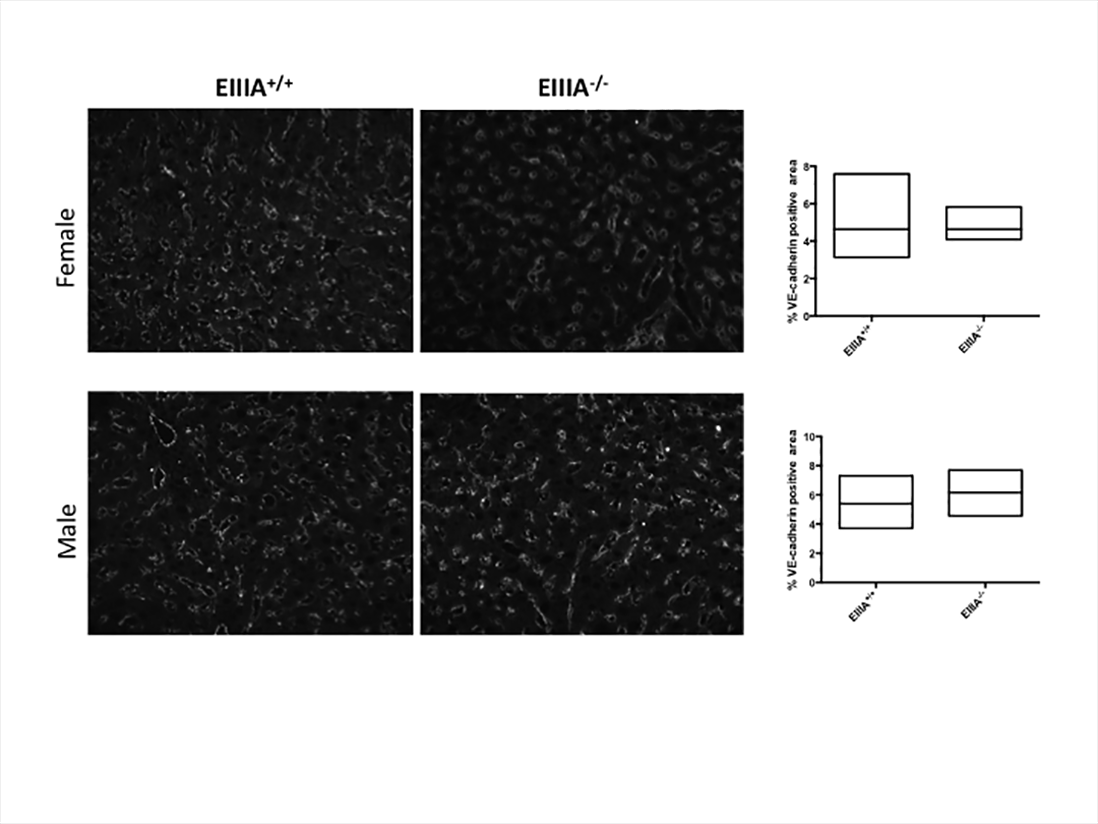

Supplement: S8 Fig — Frozen liver sections taken at day 5 after PHx were stained for VE-cadherin (white). Wild type livers from female and male mice showed comparable staining for VE-cadherin (A, C) compared to livers from EIIIA-cFN null mice (B, D). Scale bar, 50 μm. (E, F) Quantification = minimum to maximum % VE-cadherin-positive area measurements with line at mean, EIIIA+/+ (n = 8; 4 female, 4 male), EIIIA-/- (n = 8; 4 females, 4 males). (TIF) [file pone.0163737.s008.tif]

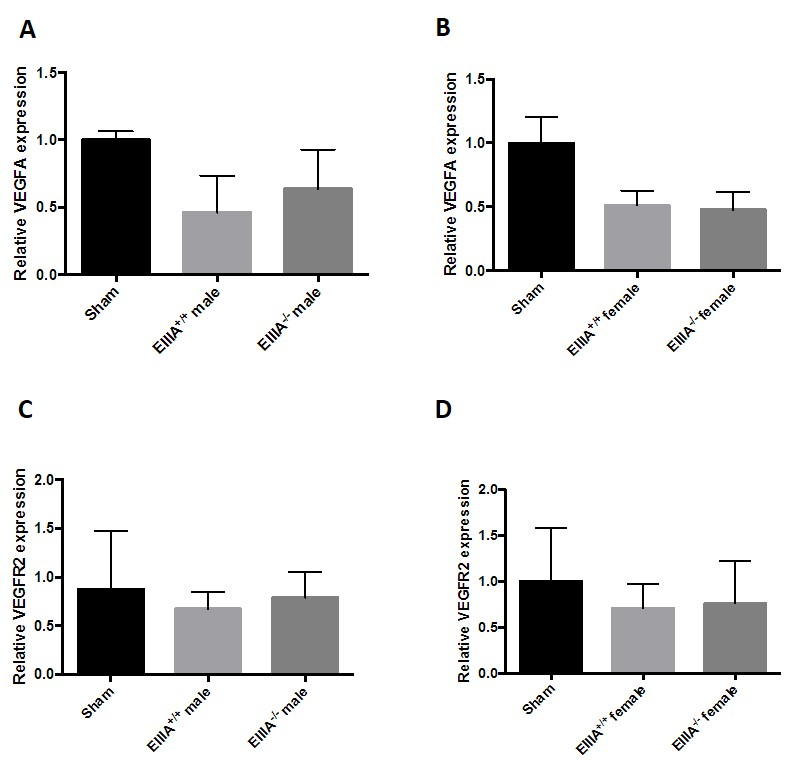

Supplement: S9 Fig — Total RNA was purified from liver lysates at day 2 following PHx and the expression of (A, B) VEGFA and (C, D) VEGFR2 was determined by qRT-PCR and normalized to the expression of tbp. Sham = 7, EIIIA+/+ (n = 11, 5 female, 6 male), EIIIA-/- (n = 12, 5 female, 7 male). (TIF) [file pone.0163737.s009.tif]

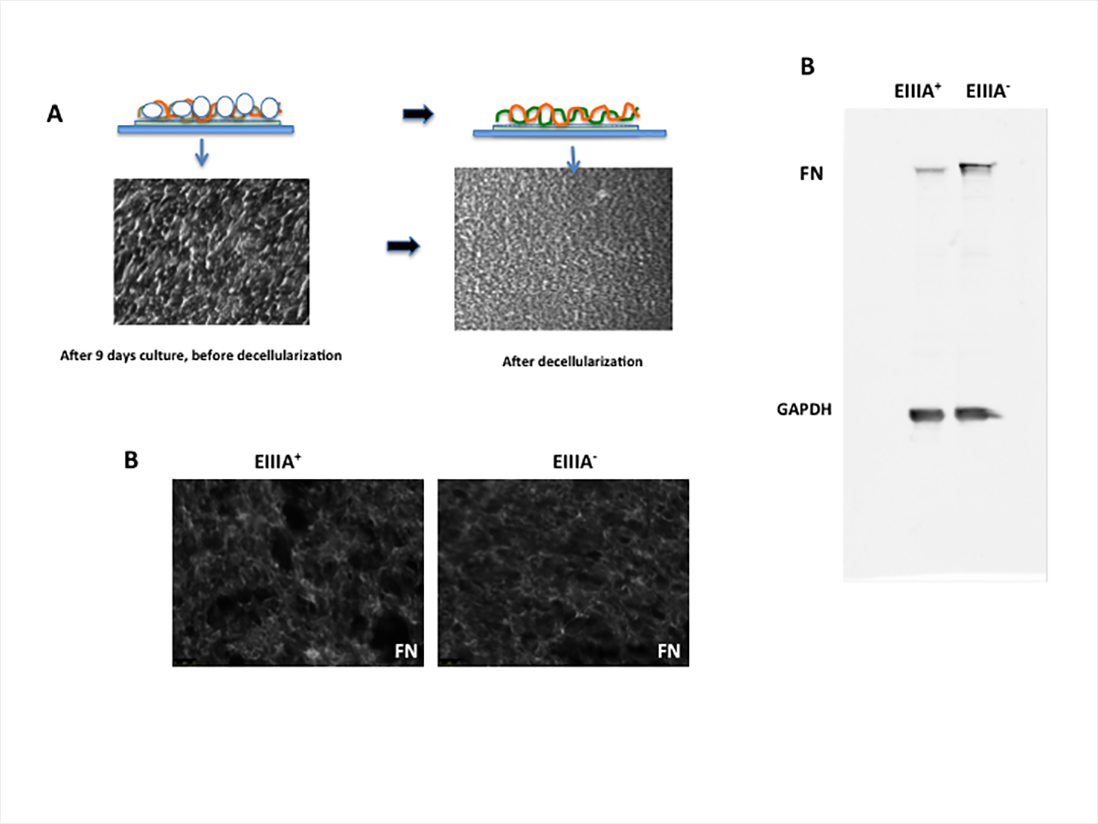

Supplement: S10 Fig — (A) CHO cells overexpressing EIIIA+ and EIIIA- cFNs were cultured for 8 days with ascorbic acid as shown in schema. (B) Immunostaining of matrices for total fibronectin. (C) Immunoblot of proteins from decellularized EIIIA+ and EIIIA- matrices, probed for total fibronectin. Loading control GAPDH. (TIF) [file pone.0163737.s010.tif]

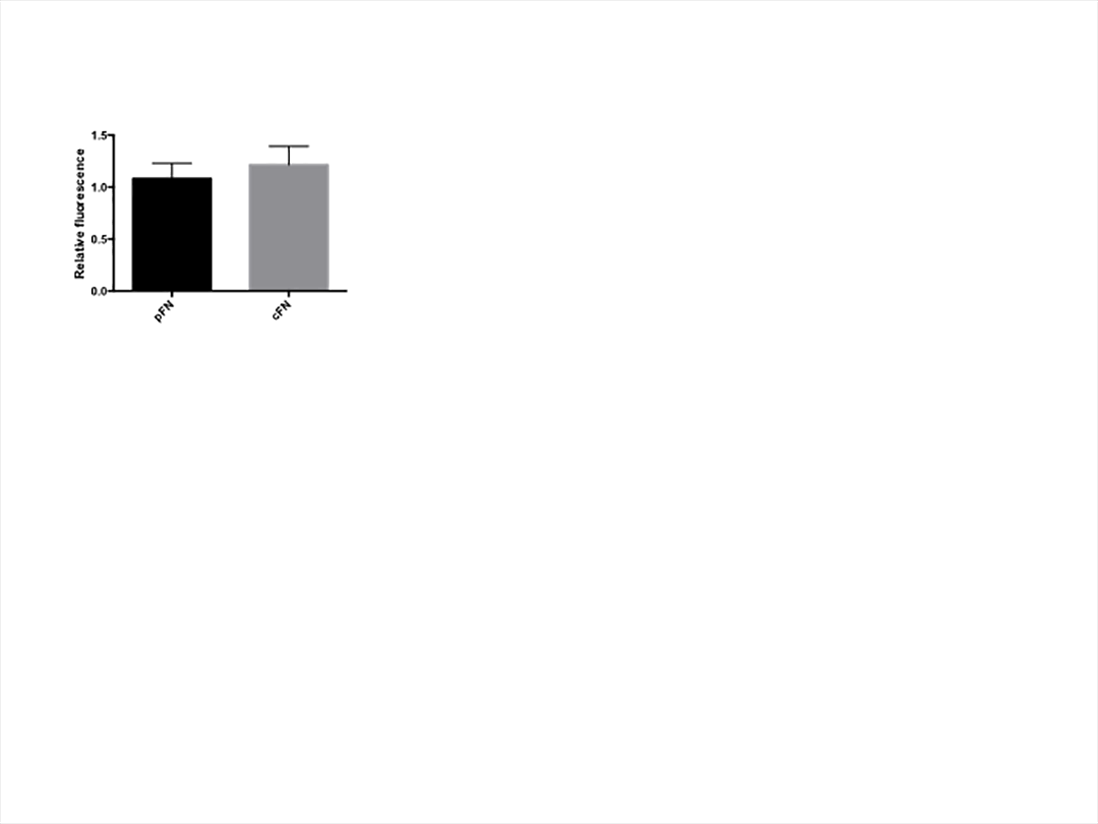

Supplement: S11 Fig — TSECs were plated on cFN- or pFN-coated transwell inserts for 19 hours. Cells were then stained with calcein and mean fluorescence intensity on the underside of the filter was measured. Graph shows mean +/- SD. (TIF) [file pone.0163737.s011.tif]
