# Supplementary material for: Fibronectin Extra Domain A Promotes Liver Sinusoid Repair following Hepatectomy
Source: PLoS One. 2016 Oct 14;11(10):e0163737. doi: 10.1371/journal.pone.0163737 (PMC5065221; doi:10.1371/journal.pone.0163737)
Supplement: S1 Table — Primer pairs were designed using Integrated DNA Technologies SciTools Real-Time PCR software. (DOCX) [file pone.0163737.s012.docx]

**S1 Table: RT-PCR primer sequences**

| **Protein name** | **Forward primer 5' - 3'** | **Forward primer 3' - 5'** |
| --- | --- | --- |
| Fibronectin (total) | CTTTGTGGTCTCATGGGTCTC | AGCAGGTCAGGAATGTTCAC |
| EIIIA cFN | AGTCAGTGTGGTTGCCTTG | CTGAACACTGGGTGCTATCC |
| VEGFA | GGCAGCTTGAGTTAAACGAAC | TGGTGACATGGTTAATCGGTC |
| VEGFR2 | ATAGAAGGTGCCCAGGAAAAG | TCTTCAGTTCCCCTTCATTGG |
| HGF | GAGTCTGAGTTATGTGCTGGG | ACGACCAGGAACAATGACAC |
| TBP | AAGAAAGGGAGAATCATGGACC | GAGTAAGTCCTGTGCCGTAAG |
| α_4_ integrin subunit | ATAAAGGCAAAGAGGTCCCAG | CGTCAGAAGTCCCATTAGAGAAG |
| α_5_ integrin  subunit | GCTACTTGGGATACTCTGTGG | GTTGTAGAGGGAGTGGATGTC |
| α_9_ integrin  subunit | TCAACATCACAGCACCTCAG | AGCCGTCAGATTGTAGTTCAG |
| α_V_ integrin  subunit | ACAGATGCAGTGTGAGGAAC | AAATGGTGATGGGAGTGAGC |
| Ang 2 | GCTGGTGAAGAGTCCAACTAC | GATGCTACTTATTTTGCCCGC |
